# Data analysis on the level of exposure to pollutions in industrial zone: A case study of Ewekoro and Ota Township

**DOI:** 10.1016/j.dib.2018.05.078

**Published:** 2018-05-18

**Authors:** G.U. Fayomi, O. Wusu, S.E. Mini, O.S.I. Fayomi, O. Kilanko

**Affiliations:** a Strategic Business Unit, Covenant University, Ota, Ogun State, Nigeria; b Department of Sociology and Centre for Environmental Studies and Sustainable Development, Lagos State University, Ojo, Nigeria; c Department of Geography, University of South Africa, Johannesburg, South Africa; d Department of Mechanical Engineering, Covenant University, P.M.B. 1023, Ota, Nigeria; e Department of Chemical, Metallurgical and Materials Engineering, Tshwane University of Technology, Pretoria, South Africa

**Keywords:** Industrial zone, Pollution, Emission, Eco-system

## Abstract

This study focused on a comparative analysis of exposure to pollution in Ota and Ewekoro Township where we have concentration of industries that emits pollutant to the air. This was with a view to proffer solution to the negative effects of industrial activities on residents within industrial location. The study involved empirical observation and interview of residents. About 652 questionnaires were administered randomly on the residents. Analysis involved descriptive statistical tools including chi-square techniques. The results suggest that air pollution was most frequently reported in Ewekoro and Ota and this can help in the prediction of stringent factor in which industrial activities could pose to society.

**Table:** 

Subject area	*Environmental Science and Engineering*
More specific subject area	*Pollution and urban system*
Type of data	*Table, image*
How data was acquired	For the purpose of these research works, a systematic sampling technique was used. This involved selecting every 10houses in Major Street and every 5 houses in Minor Street. In Ota, 20 major street and 13minor street was selected making a total of 320 for Ota and 332 questionnaire administered for Ewekoro township.
Data format	Raw, Analyzed
Experimental factors	A number of 68 streets were surveyed in the two study area where every 10 houses were selected at random in long streets and 5 houses in short street to make a total of 652 houses as the sample size.
Experimental features	The primary sources of data collection in this study involves the use of various methods of data collection of information such as the use of questionnaire, direct interviews, personal observation and the use of photographs. The data sources were utilized to ensure comprehensive exploration of this study.
Data source location	Ewekoro and Ota industrial zone
Data accessibility	Data are available within this article

**Value of the data**

• The given data will show author in the field of environmental management and urban renewal the trend of pollution as it relate to industrial activities.
• The data obtained can be used as inference to understand clearly the percentage distribution of respondents by socio-economic and physical characteristics
• The data can be used to examine the relationship between the different levels of disposition to various environmental hazards.

## 1 Data

In an attempt at appreciating the respondents perception on pollution generated from the industries, residents were asked to indicate their perceived causes in the study settings. Table 1 shows the percentage distribution of respondents by selected characteristics as reported by Refs. [1], [2], [3]. This include percentage distribution of respondent by age, sex, religion, educational qualification, average monthly income, type of property occupied and approximate distance between the factory site and individual houses. Age group 25–29 years has the highest proportion of the respondents in Ota (22%) and Ewekoro (31%). While male respondents were in majority in Ota (59%) female were in majority in Ewekoro (53%).

**Table 1:** Data on percentage distribution of respondents by socio-economic and physical characteristics.

	Ota	%	Ewekoro		Total	Percentage
**Age distribution**						
15–19	20	6	19	6	39	6
20–24	54	17	43	13	97	15
25–29	66	22	102	31	168	26
30–34	57	18	83	25	140	21
35–39	26	8	32	9	58	9
40–44	31	9	15	5	46	7
45+	45	14	11	3	56	8
No response	21	6	27	8	48	7
Total	320	100	332	100	652	100
**Sex distribution**						
Male	191	59	141	46	345	53
Female	118	36	175	53	293	45
No response	11	4	3	0.9	14	2
Total	320	100	329	100	652	100
**Religion**						
Catholic Christians	124	38	199	59	323	50
Non Catholic Christians	162	51	60	18	222	34
No response	34	11	73	22	107	16
Total	320	100	332	100	652	100
**Educational qualification**						
SSCE	111	35	91	27	202	31
Tertiary Education	181	56	227	68	408	63
No response	28	8	14	4	42	6
Total	320	100	332	100	652	100
**Monthly income**						
5000–15,000	49	15	24	7	73	11
16,000–25,000	69	22	80	24	149	23
26,000–35,000	67	21	75	23	142	22
36,000–45,000	34	11	55	16	89	14
46,000–55,000	16	5	24	7	40	6
56,000–65,000	8	3	5	2	13	2
66,000+	37	12	26	8	63	9
No response	40	13	43	13	83	13
Total	320	100	332	100	652	100
**Type of property**						
Tenement apartment	135	42	199	59	334	52
2b/r flat	88	27	109	33	197	31
Others	77	24	20	6	97	14
No response	20	6	4	2	22	3
Total	320	100	332	100	652	100
**Approximate distance in metre**						
Less than 100 m	79	29	57	17	136	21
100–549 m	140	44	168	51	316	48
550–999 m	32	10	29	6	61	9
1 km+	50	16	63	18	113	17
No response	19	6	15	5	34	5
Total	320	100	332	100	652	100

**Fig. 1:**
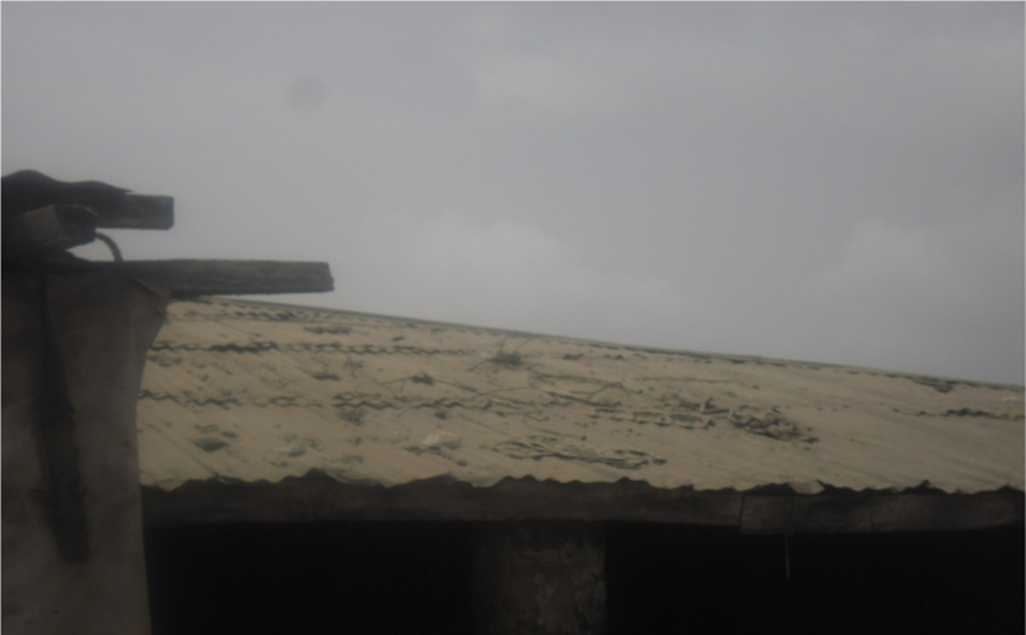
Roof cover with cement dust.

**Fig. 2:**
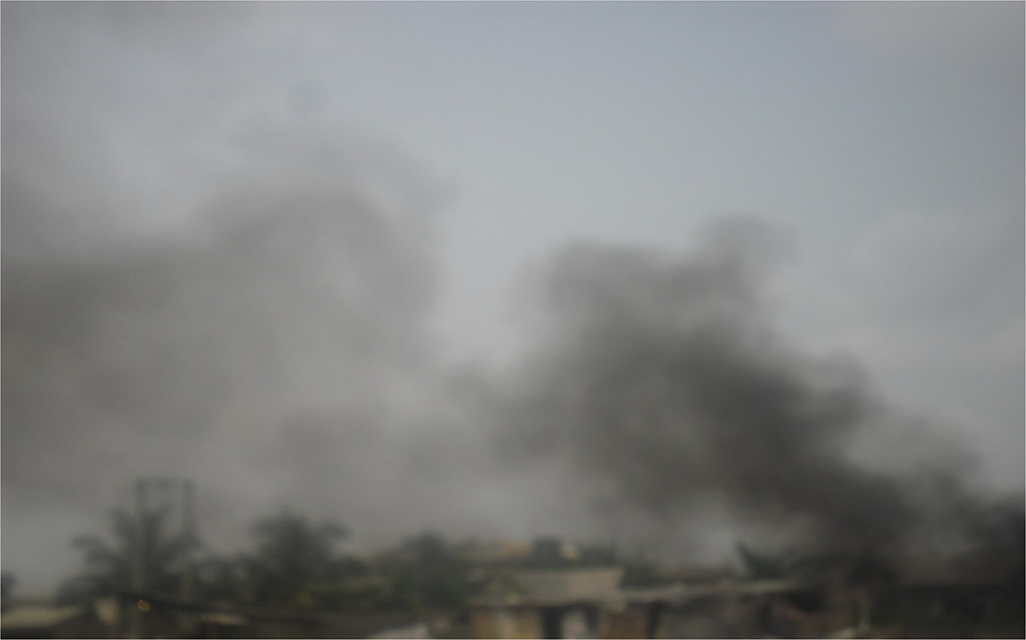
Smoke released into the air from industrial activities in Ota industrial estate.

More so analysis shows that 89% of the respondents reported industrial causes as the major sources of pollution within their neighborhood while 7% of the study sample indicated other causes as presented in Table 2, Table 3. Respondents were asked to rate the various types of pollution in an attempt to confirm the different levels of disposition to various environmental hazards. The analysis in Table 4 shows that air pollution rated high prevalence with 50% in Ota and 54% in Ewekoro compared to noise pollution with 36% and 22% high prevalence. Water pollution has low rate with 5% in Ota and 11% in Ewekoro. The photo view of the rapid industrialised activities within the region is presented in Fig. 1, Fig. 2.

**Table 2:** Data on percentage distribution of respondents by causes of pollution in Ota and Ewekoro.

Causes of pollution	Ota No	%	Ewekoro No	%	Total	Percentage
Other causes	39	13	3	0.9	42	7
Industrial causes	263	83	321	95	584	89
No response	11	4	15	4	26	4
Total	320	100	332	100	652	100

**Table 3:** Data on the percentage distribution of respondents by types of pollution in Ota and Ewekoro.

Common types of pollution	Ota	%	Ewekoro	%	Total	Percentage
Air pollution	154	48	172	52	326	50
Noise pollution	52	16	113	34	165	25
Water pollution	11	4	33	9	44	7
Air and noise pollution	64	20	9	3	73	11
All the above	28	8	2	0.6	30	5
No response	11	4	3	0.9	14	2
Total	320	100	332	100	652	100

**Table 4:** Data on the perception of the rate of various types of pollution in Ota and Ewekoro.

Air pollution rate	Ota N	%	Ewekoro N	%	Total	Percentage
None	4	2		0.3	5	0.7
Low	23	7	15	5	38	6
Medium	79	25	81	24	160	25
High	160	50	180	54	340	52
No response	54	16	55	16	109	17
Total	320	100	332	100	652	100
Noise pollution rate						
None	6	2	2	0.6	8	2
Low	20	7	13	4	33	5
Medium	92	28	139	42	231	35
High	117	36	73	22	190	29
No response	85	26	105	31	190	29
Total	320	100	332	100	652	100
Water pollution rate						
None	49	15	7	2	56	8
Low	76	23	69	21	145	22
Medium	45	14	64	19	109	16
High	17	5	57	17	74	11
No response	133	41	135	41	268	41
Total	320	100	332	100	652	100

## 2 Experimental design, materials and methods

Quantitative data collection method was used for this study. This strategy includes the use of questionnaire, direct interviews, personal observation and the use of photographs [3]. The data sources were utilized to ensure comprehensive exploration of the investigation. All the data collected for the purpose of this study were analysed using statistical techniques such as tabulations, bar-charts and histogram, frequency polygon, cross tabulations and photographs at univarate and bivariate levels of analyses was employed during the process of data analysis and presentation [4], [5]. Chi square was used to determine the association between the perceived level of exposure and health conditions of the inhabitants as stated in the hypothesis. The study population include men and women aged 18years and above in Ota and Ewekoro township.
