# Supplementary material for: Data analysis on the level of exposure to pollutions in industrial zone: A case study of Ewekoro and Ota Township
Source: Data Brief. 2018 May 18;19:859–64. doi: 10.1016/j.dib.2018.05.078 (PMC5997909; doi:10.1016/j.dib.2018.05.078)
Supplement: Supplementary file 1 — Supplementary material [file mmc1.docx]

***COVER LETTER/CONFLICT OF INTEREST ATTESTATION***

*11th May, 2018*

*The Editor-in-Chief*

*Data in Brief*

***Subject:***  ***NO CONFLICT OF INTEREST***

*Dear Sir,*

*This serve to notify you that the manuscript is original of the authors work and there is no conflict of interest of any kind regarding the manuscript* Data analysis on the level of exposure to pollutions in Ewekoro and Ota Township industrial zone

*Sincerely yours,*

*Dr. Gloria Fayomi*

*Strategic Business Unit*

*Covenant University
Ota,*

*Nigeria*
